# A novel UBE2A splice site variant causing intellectual disability type Nascimento

**DOI:** 10.1002/ccr3.5990

**Published:** 2022-07-11

**Authors:** Shuyuan Yan, Yanling Wang, Ying Chen, Hongxia Yuan, Xiaoni Kuang, Da Hou, Xueyi Li, Linglin Pan, Guangwen Huang, Jun He, Tuanmei Wang, Xiangwen Peng

**Affiliations:** ^1^ Changsha Maternal and Child Health Hospital Affiliated to Hunan Normal University Changsha China; ^2^ Hunan Provincial Maternal and Child Health Care Hospital Changsha China

**Keywords:** UBE2A splice site mutation (c.241 + 1 G > a), X‐linked intellectual disability type Nascimento

## Abstract

X‐linked intellectual disability type Nascimento (XLID) is a rare disease caused by variants in the ubiquitin‐conjugating enzyme E2A gene (UBE2A). Patients with XLID have similar phenotypes, including speech impairments, severe intellectual disability, hearing loss, wide facies, synophrys, generalized hirsutism, and urogenital abnormalities. Till date, only two splice‐site variants of the UBE2A gene have been observed in patients with X‐linked ID type Nascimento. Here, we report the case of a Chinese boy with a syndrome clinically similar to XLID with speech impairment, severe intellectual disability, and moderate hearing loss. However, different characteristics were also present in the patient, including an inability to maintain his head in an upright posture. Both of the patient's palms have a single transverse palmar crease. Subsequent whole‐exome sequencing revealed a novel splice site variant in UBE2A (c.241 + 1 G > A). Our study not only expands the variant spectrum and clinical characteristics of UBE2A deficiency syndrome but also provides clinical evidence for genetic diagnoses.

## Graphical abstract

Here, we report the case of a boy with a novel splice site variant in UBE2A. In addition to intellectual disability, we found novel characteristics, such as bone growth retardation in the fetus. Cardiac malformations were found in the fetal and neonatal period but disappeared as the patient grew older.

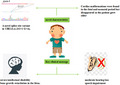

## 1 INTRODUCTION

X‐linked ID type Nascimento (XLID), characterized by a syndromic intellectual disability due to gene variants on the X chromosome, has received great attention due to the high incidence rate of intellectual disability in males.^1^, ^2^, ^3^, ^4^, ^5^ According to recent reports, the X‐chromosome comprises only approximately 5% of the human genome but accounts for approximately 15% of the genes currently known to be associated with intellectual disability.^6^

Ubiquitin‐conjugating enzyme E2 (UBE2A) is involved in the proteasome pathway for protein degradation and DNA repair^7^ and is located on Xq24.^8^, ^9^, ^10^ UBE2A contains 6 exons, and the protein contains 119 amino acids. UBE2A deficiency syndrome, also known as X‐linked ID type Nascimento (MIM #300860), was first described by Nascimento in 2006 and was characterized clinically by a pronounced delay in psychomotor development, wide facies, synophrys, generalized hirsutism, a horizontal eyebrow, and urogenital abnormalities.^11^, ^12^ Since then, two splice‐site variants in UBE2A, seven missense variants in UBE2A, and four larger deletions in UBE2A have been found.^7^, ^13^ However, there have been few reports of UBE2A splice site variants in China.

Here, we report the case of a Chinese patient diagnosed with XLID with a novel UBE2A splice site mutation (c.241 + 1 G > A) with a few unique clinical features. Identifying novel variants of UBE2A in XLID will help prevent disability and provide more opportunities to explore the molecular basis of intellectual disability.

### 1.1 Ethics compliance

Written informed consent was obtained from the patient's parents, and all of the procedures were reviewed and approved by the Ethics Committee of Changsha Hospital for Maternal and Child Health Care at Hunan Normal University (Hunan, Changsha, China).

## 2 CASE PRESENTATION

**FIGURE 1:**
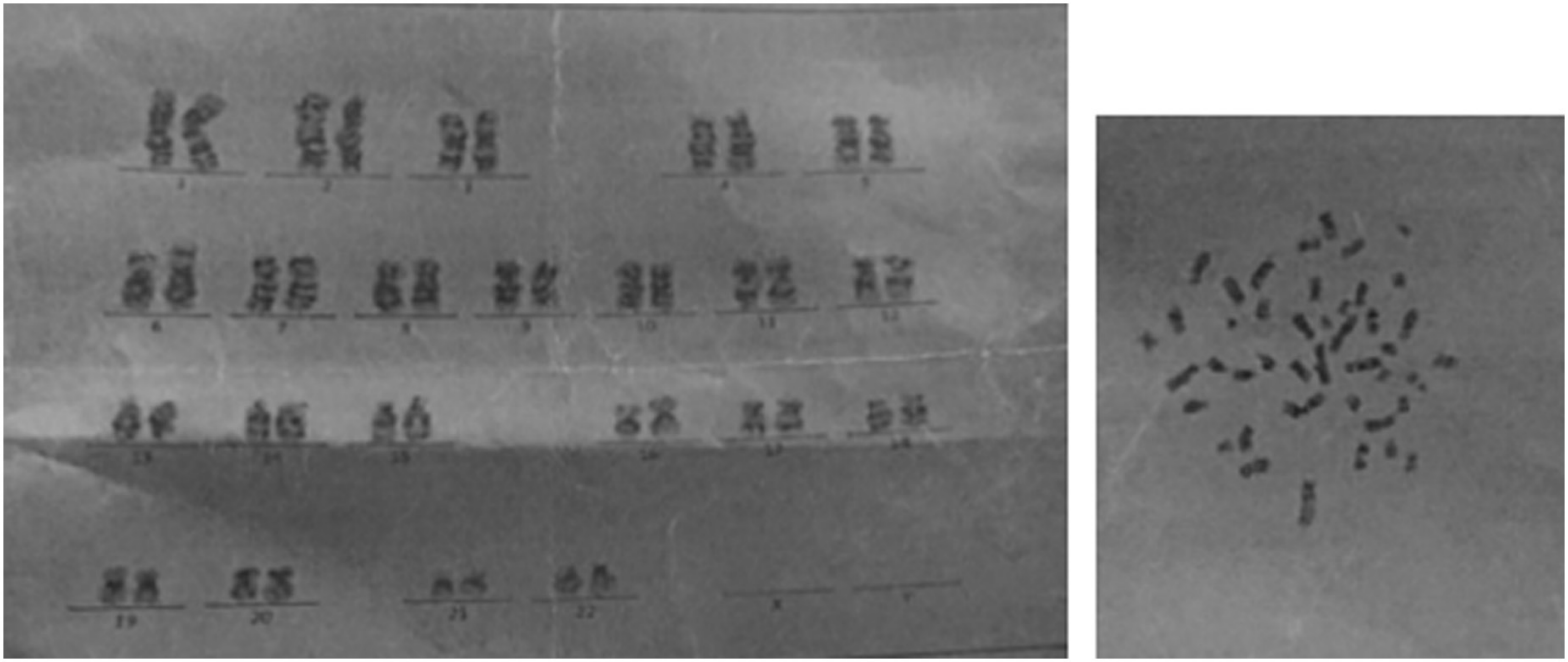
Karyotype analysis showed no abnormalities

The proband was born on September 18, 2018; a boy, G1P1, 35 weeks 4 days gestation, birthed by cesarean section due to fetal distress, with a birth weight of 2.15 kg, and an unknown Apgar score. A history of intrapartum hypoxia or asphyxia was denied. At a gestational age of 34 weeks and 4 days, Doppler ultrasound showed that the femur and humerus were at a size expected for approximately 30 weeks of gestational age, the internal diameter of the fetal aortic arch and isthmus was slightly narrow, the umbilical vein was abnormal, the S/D ratio of the umbilical vein was higher than the RI value, and the S/D and RI values of the middle cerebral artery were lower. Neither numerical nor gross structural chromosomal abnormalities were found (Figure 1). A SNP (Affymetrix CytoScan 750 K Array) gene chip analysis revealed no pathogenic microdeletion or microduplication aberrations in the whole genome.

**FIGURE 2:**
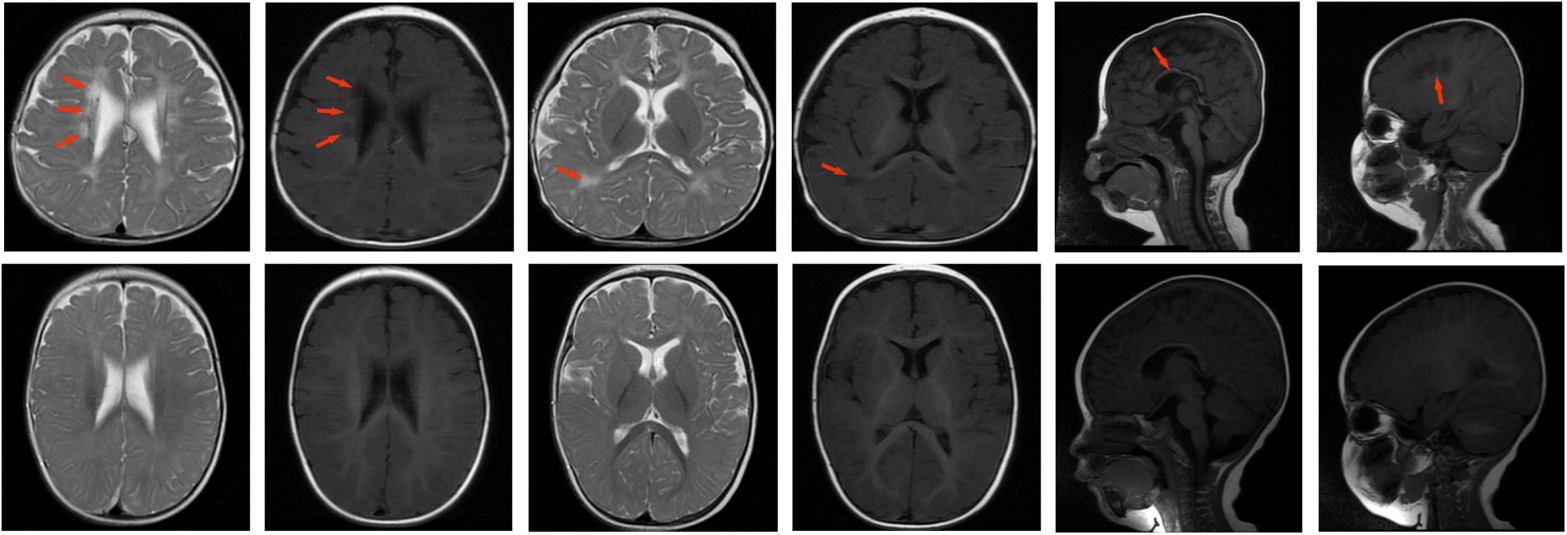
MRI scan of the patient's brain showed abnormal signal changes in the deep white matter area near the lateral ventricle and a mild delay of myelination compared with a healthy child at the age of 7 months

On September 18, 2018, the patient was hospitalized for 28 days for neonatal treatment at the Second Affiliated Hospital of Xiangya as a result of “premature birth and intrauterine distress” (the specific diagnoses and treatments administered are unknown). The patient remained unable to maintain his head in an upright posture from 7+ months to 40 months, and movement training was initiated. We noticed that both of the patient's palms had a single transverse palmar crease and that his intellectual development was significantly behind that of children of the same age; therefore, we considered the diagnosis of developmental delay and genetic mutation. Brain MRI (April 3, 2019) showed abnormal signal changes in the deep white matter area adjacent to the lateral ventricle (symmetrical distribution of patch‐like, slightly longer T1 and slightly longer T2 signal changes can be seen in the deep white matter area beside the lateral ventricles of the bilateral cerebrum, with low signal on DWI and slightly low signal on T2 FLAIR) (Figure 2). The patient's rehabilitation program has continued till date.

**FIGURE 3:**
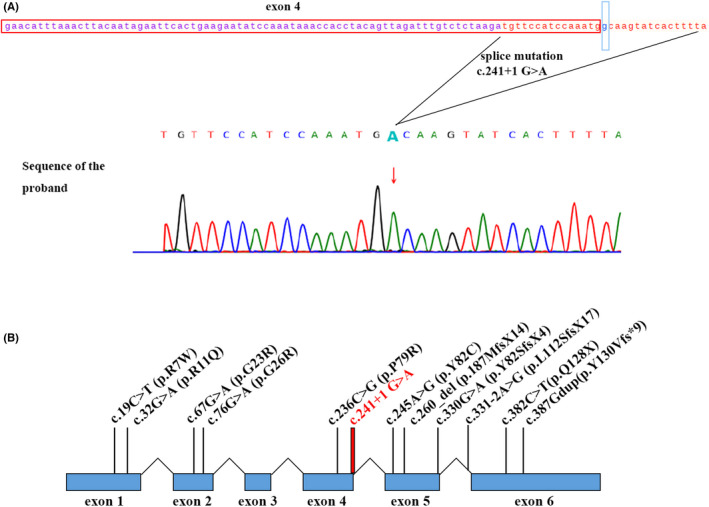
Variant sequencing of the proband. (A) One nucleotide (241 + 1 G > A, in blue) near exon 4 (in red frame) was modified, likely influencing UBE2A gene splicing. UBE2A DNA (in red sequencing). (B) Schematic of the UBE2A gene, showing the position of the newly identified (in red) and previously published variants (in black). Reference sequence for the UBE2A gene: NM_003336.3

Whole‐exome sequencing (WES) detection revealed the following: UBE2A:NM_003336.3:exon4:c.241 + 1G > A: variant: pathogenic (PVS1 + PM2 + PP3) (Figure 3). Strong evidence of pathogenicity (PVS1) was present as the mutation identified occurs in the splicing region and may lead to altered protein function. There was moderate pathogenic evidence (PM2) as the mutation is listed in the Berry gene Chinese population‐specific database “Shenzhou Genome Database” and the Human Exon Database (ExAC), but not in the Reference Population Thousand Genomes (1000G) and Population Genome Mutation Frequency Database (gnomAD). The supporting pathogenic evidence (PP3) included the following: a conservative prediction by GERP, showing that this locus is evolutionarily conserved and has the potential for functional impact, and a protein function prediction by CADD and DANN, indicating that the mutation is harmful.

According to a public database query, UBE2A gene mutations (OMIM:312180) can cause X‐linked recessive, X‐linked syndromic, and Nascimento‐type intellectual disability (OMIM: 300860, Budny et al. al., 2010). X‐linked intellectual disability syndrome Nascimento type mainly manifests as intellectual disability, language delay, midface dysplasia, and small feet.

**FIGURE 4:**
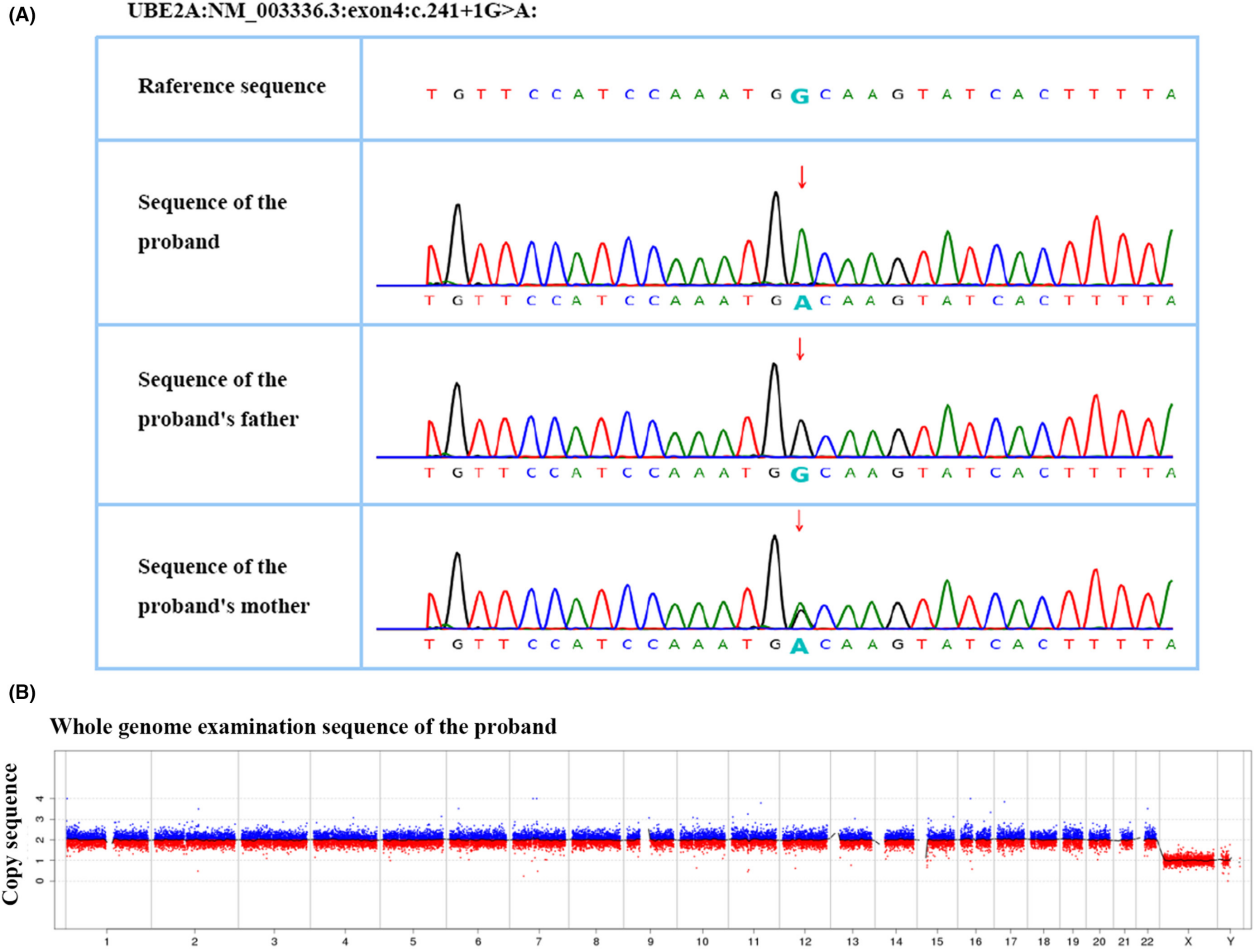
Sequencing and DNA analysis of the proband's family members (A) c.241 + 1 G > A. (B) No chromosomal aneuploidy or known, definite pathogenic genomic copy number variants (CNVs) greater than 100 kb were detected in this sample

The results of next‐generation sequencing showed that the proband had a hemizygous UBE2A mutation (c.241 + 1G > A), the mother had a heterozygous mutation, and the father had no mutation; therefore, the mutation was inherited from the phenotypically normal mother (Figure 4A).

The CNV‐seq test results revealed no chromosomal aneuploidy or known, definite pathogenic genomic copy number variations (CNVs) above 100 kb (Figure 4B).

The family denied any similar medical history in the family and any genetic metabolism‐related family medical history.

**FIGURE 5:**
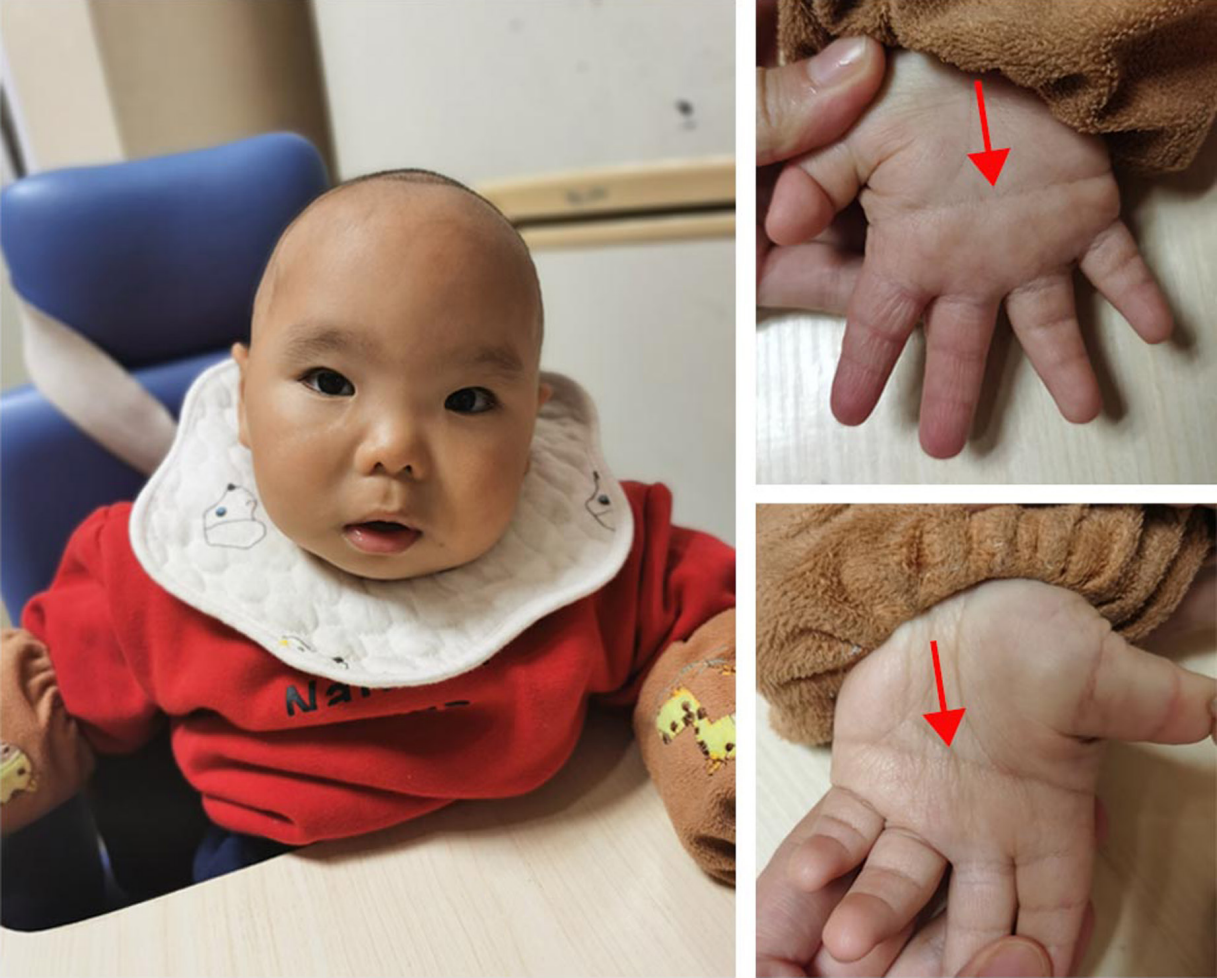
Clinical characteristics of the patient. (A) Flat face; round face; wide‐set eyes; the red arrows indicates both palms have a single transverse palmar crease

A physical examination conducted at the age of 7 months revealed that the patient's height was 62.5 cm, his head circumference was 40.0 cm, and his weight was 6.9 kg. The patient presented with a short stature, flat face, wide eye spacing, and a single transverse palmar crease. The patient's face was round, and his upper and lower limbs were short (Figure 5A–E). Cardiopulmonary auscultation showed no obvious abnormalities.

A professional examination was conducted at the child health department, and the patient's mental performance was determined to be normal, his responses average, and his follow‐up and listening abilities were good. However, the patient insensitive to teasing, little active pronunciation and was prone to crying. He was unable to hold his head up for more than 15 s, was happy leaning back, held his head up at an angle less than 45°, demonstrated poor elbow support, was unable to roll over, and showed poor lower body weight support. The muscle tone of the limbs was low, and the tendon reflexes were slightly weakened.

In the auxiliary examination, during the patient's hospitalization in the neonatal department, echocardiography showed patent ductus arteriosus, congenital ventricular septal defect and patent foramen ovale. A hearing test (April 3, 2019) revealed the following: 40 dnBl/50 dnBl. On April 24, 2019, re‐examination at our hospital revealed no obvious abnormality. An X‐ray of the hip joint (December 9, 2019) at our hospital showed no obvious abnormalities, and repeated thyroid function, routine blood tests, and liver and kidney function tests showed no obvious abnormalities.

A Gesell test (May 18, 2020) was conducted and revealed the following: 29 points for adaptability, 30 points for gross motor, 34 points for fine motor, 46 points for language, and 47 points for personal‐social.

Till date, for treatment, the child has been administered exercise training, EEG, EMG, intermediate frequency therapy, biofeedback, occupational therapy, language training, and other interventions in our department.

Currently, the child is able to walk with assistance. He can walk several steps by himself but cannot squat or stand alone. After a fall, the patient moves into a sitting position and stands up with the assistance of an object. He can say common names such as mom, dad, and grandma, and he can name common items, with a vocabulary of approximately 10 words. His word comprehension is dominated by nouns, approximately 30 words. He can understand simple commands. He is able to pinch pills with his thumb and index finger, but his fingertip pinching is poor. He is able to build with 2 building blocks, uses the index finger to point to objects, and can hold a pen to doodle; however, he cannot match a shape to a board, cannot self‐feed with a spoon, and requires assistance with putting on and taking off his clothes. His limb muscle tone remains slightly low. The patient is uncooperative in muscle strength examinations, but the tendon reflexes can be elicited by Gesell's test (December 2, 2021), and the most recent findings are as follows: 35 points for adaptability, 29 points for gross motor, 28 points for fine motor, 47 points for language, and 28 points for personal‐social.

## 3 DISCUSSION

In the present study, we used whole‐exome sequencing to identify a novel UBE2A splice site variant (c.241 + 1 G > A) in a Chinese proband with X‐linked intellectual disability. This is the second case report of this syndrome associated with novel splice site variants in China, to the best of our knowledge.

Notably, we report novel molecular and clinical data of this patient with intellectual disability. Till date, only two splice‐site variants in UBE2A, seven missense variants in UBE2A, and four larger deletions in UBE2A have been reported.^7^, ^12^, ^13^, ^14^, ^15^, ^16^, ^17^, ^18^, ^19^, ^20^, ^21^ In addition, all probands with UBE2A deficiency syndrome have similar phenotypes, including characteristic facial appearance, speech anomalies, and intellectual disability.^18^, ^22^ However, the clinical presentation of our proband was novel, including a deep single transverse palmar crease and an inability to hold the head erect, which has not been described in previous patients.

In addition, the phenotypes of all reported probands with UBE2A variant syndrome and the patient described in the present study were analyzed, and three UBE2A variant syndrome patient groups could be classified: (1) those with larger deletions (largedel), (2) those with missense variants (miss), and (3) those with splice site variants (splice) (Table 1). All patients were males and showed severe intellectual disability and speech impairment.

**TABLE 1:** Clinical and molecular data of patients with UBE2A Deficiency Syndrome due to missense mutation of UBE2A (Each in a Separate Column), as well as of patients with splice site mutation (splice), each type of mutation are summarized in a separate column—larger deletions (largedel), missense mutation (miss), and splice site mutation (splice) in UBE2A

References		Dingyuan Ma et.al (2019)	Giugliano et al. (2018)		Nascimento et al.(2006)		Budny et al. (2010)					Haddad et al. (2013)			Czeschik et al. (2013)	Tsurusaki et al. (2017)	Weimin Jia et al. (2019)	de Leeuw et al; Honda et al; Thunstrom et al; Czeschik et al;		
Patient	1	1	III.3	III.8	1	2	IV:4	V:2	IV:13	III:12	IV:3	III‐5	III‐6	III‐7	5	1	II:3			
General																				
Type of mutation	Splice	Splice	Splice		Miss		Miss					Miss			Miss	Miss	Miss	Largedel	Miss	Splice
Number of different mutation																		4	7	3
Number of patients																		9	13	4
Gender	M	M	M	M	M	M	M	M	M	M	M	M	M	M	M	M	M	M	M	M
Ethnicity	Chinese	Chinese	Caucasian		Brazilian		Caucasian					Caucasian				Japanese	Chinese			
Mutation	c.241 + 1G > A	c.331‐2A > G (p.L112SfsX17)	c.330G > A (p.Y82SfsX4)		c.382C > T		c.67G > A (p.G23R)				c.32G > A (p.R11Q)	c.19C > T (p.R7W)			c.236C > G (p.P79R)	c.76G > A (p.G26R)	c.245A > G (p.Y82C)			
Gestational weeks	35 + 4 W	NA	39	40	35	35	NA	39	40	NA	39	NA	NA	NA	40	39	NA			
Birth weight (g)	2150	NA	3970	3150	2405	3200	NA	3500	5500	NA	3600	NA	NA	NA	2800	2355	NA			
Birth height (cm)	NA	NA	36	35	48	50	NA	NA	NA	NA	NA	NA	NA	NA	NA	46	NA			
Development																				
Inability to maintain his head in upright position	+	NA	NA	NA	−	−	NA	NA	NA	NA	NA	NA	NA	NA	NA	NA	NA	0/0	0/0	1/1
Speech impairment	+	+	+	+	+	+	+	+	+	+	+	+	+	+	+	+	+	9/9	13/13	4/4
Intellectual disability	+	+	+	+	+	+	+	+	+	+	+	+	+	+	+	+	+	9/9	13/13	4/4
Heart defects	−	NA	−	NA	+	+	NA	NA	NA	NA	NA	NA	NA	NA	−	+	NA	9/9	3/4	0/2
White matter abnormalities	+	+	NA	NA	+	NA	NA	NA	NA	NA	NA	NA	−	+	−	+	−	6/7	3/6	2/2
Dysmorphic features																				
Synophrys	−	−	+	+	+	−	+	+	+	+	+	+	+	+	+	+	+	6/9	12/13	2/4
Ocular hypertelorism	+	−	+	+	+	+	−	−	−	−	−	NA	NA	NA	−	−	+	8/9	3/10	3/4
Wide face	+	+	+	+	NA	NA	−	−	+	+	−	NA	NA	NA	−	+	NA	0/0	3/7	4/4
Upslanting palpebral fissures	−	−	−	−	+	+	NA	NA	NA	NA	NA	−	−	−	−	−	+	7/9	3/8	0/4
Small feet	+	NA	−	−	+	+	−	−	+	+	−	−	−	−	−	NA	−	5/8	4/12	1/3
Generalized hirsutism	−	NA	+	+	+	−	NA	NA	+	+	+	+	+	+	+	+	+	4/9	10/11	2/3
Hearing loss/impairment	+	NA	−	−	+	NA	NA	NA	NA	NA	NA	NA	NA	NA	+	NA	NA	2/8	2/2	1/3
Genital anomalities																				
Small penis	+	NA	+	NA	+	+	−	+	+	+	+	−	+	+	−	NA	−	7/9	8/12	2/2

*Note*: Patients with missense mutation (miss) and splice site mutation (splice) include patient 1 (described here. The patients with Larger Deletions (largedel)reported by de Leeuw et al (2010), Hoddad et al. (2010), Czeschik et al. (2013), Thunstrom et al. (2015). Each Type of Mutation are Summarized in a Separate Column—Larger Deletions (largedel), missense mutation (miss), and splice site mutation (splice), number of affected individuals with available clinical data.

Abbreviation: NA, not available.

In our case, cardiac malformations were found in the fetal and neonatal period but disappeared as the patient grew older. This observation is different from that of other individuals with UBE2A variants with heart defects.^7^, ^11^, ^12^, ^17^, ^20^, ^23^ According to recent reports, four other patients did not have upslanting palpebral fissures. Although these analyses were based on a small number of patients and, therefore, it is difficult to draw valid conclusions, it is worth noting that this observation suggests that individuals with UBE2A splice site variants might not be at increased risk for upslanting palpebral fissures and heart defects. Conversely, individuals with UBE2A splice site variants might be at an increased risk for wide facies, white matter abnormalities and a small penis.

In this boy, the clinical presentation of this new phenotype included shorter limbs. At a gestational age of 34 + 4 W, a four‐dimensional color Doppler ultrasound indicated that the femur and humerus were of a size expected for a fetus at a gestation age of approximately 30 W, which may be a new phenotype of UBE2A gene variants.

In conclusion, this report describes novel splice site variants (c.241 + 1 G > A) in the UBE2A gene resulting in an aberrant appearance and severe intellectual disability in a Chinese proband. The patient was found to have a novel clinical appearance, including a deep single transverse palmar crease and inability to hold the head erect. Our report expands the variant spectrum and clinical characteristics of UBE2A deficiency syndrome (also called XLID) and may provide clinical evidence for genetic diagnoses.

## AUTHOR CONTRIBUTIONS

Jun He, Shuyuan Yan, Yanling Wang, Ying Cheng, Hongxia Yuan, Xiaoni Kuang, Da Hou, Xueyi Li, Linglin Pan, Guangwen Huang, and Jun He involved in conceptualization, data curation, and formal analysis. Tuanmei Wang involved in funding acquisition. Xiangwen Peng performed writing—review and editing.

## CONFLICTS OF INTEREST

The authors have no conflicts of interest to disclose.

## ETHICAL APPROVAL

The Ethics Committee of Changsha Maternal and Child Health Hospital approved this study.

## CONSENT

Written informed consent was obtained from the parent's patient to publish this report in accordance with the journal's patient consent policy because the patient is a child.

## Data Availability

All data, models, and codes generated or used during the study appear in the submitted article.

## References

[1] Piton A , Redin C , Mandel JL . XLID‐causing mutations and associated genes challenged in light of data from large‐scale human exome sequencing. Am J Hum Genet. 2013;93(2):368‐383. 23871722 10.1016/j.ajhg.2013.06.013 PMC3738825

[2] Lee YR , Khan K , Armfield‐Uhas K , et al. Mutations in FAM50A suggest that Armfield XLID syndrome is a spliceosomopathy. Nat Commun. 2020;11(1):3698. 32703943 10.1038/s41467-020-17452-6 PMC7378245

[3] Louie RJ , Collins DL , Friez MJ , Skinner C , Schwartz CE , Stevenson RE . Schimke XLID syndrome results from a deletion in BCAP31. Am J Med Genet A. 2020;182(9):2168‐2174. 32681719 10.1002/ajmg.a.61755

[4] Ziats CA , Schwartz CE , Gecz J , et al. X‐linked intellectual disability: phenotypic expression in carrier females. Clin Genet. 2020;97(3):418‐425. 31705537 10.1111/cge.13667

[5] Pravata VM , Omelkova M , Stavridis MP , et al. An intellectual disability syndrome with single‐nucleotide variants in O‐GlcNAc transferase. Eur J Hum Genet. 2020;28(6):706‐714. 32080367 10.1038/s41431-020-0589-9 PMC7253464

[6] Neri G , Schwartz CE , Lubs HA , Stevenson RE . X‐linked intellectual disability update 2017. Am J Med Genet A. 2018;176(6):1375‐1388. 29696803 10.1002/ajmg.a.38710 PMC6049830

[7] Thunstrom S , Sodermark L , Ivarsson L , Samuelsson L , Stefanova M . UBE2A deficiency syndrome: a report of two unrelated cases with large Xq24 deletions encompassing UBE2A gene. Am J Med Genet A. 2015;167A(1):204‐210. 25287747 10.1002/ajmg.a.36800

[8] Shen JD , Fu SZ , Ju LL , et al. High expression of ubiquitin‐conjugating enzyme E2A predicts poor prognosis in hepatocellular carcinoma. Oncol Lett. 2018;15(5):7362‐7368. 29725449 10.3892/ol.2018.8189 PMC5920371

[9] Zhao Y , Alexandrov PN , Jaber V , Lukiw WJ . Deficiency in the ubiquitin conjugating enzyme UBE2A in Alzheimer's disease (AD) is linked to deficits in a natural circular miRNA‐7 sponge (circRNA; ciRS‐7). Genes. 2016;7(12):116. 10.3390/genes7120116 PMC5192492 27929395

[10] Ramatenki V , Potlapally SR , Dumpati RK , Vadija R , Vuruputuri U . Homology modeling and virtual screening of ubiquitin conjugation enzyme E2A for designing a novel selective antagonist against cancer. J Recept Signal Transduct Res. 2015;35(6):536‐549. 25316404 10.3109/10799893.2014.969375

[11] Czeschik JC , Bauer P , Buiting K , et al. X‐linked intellectual disability type Nascimento is a clinically distinct, probably underdiagnosed entity. Orphanet J Rare Dis. 2013;8:146. 24053514 10.1186/1750-1172-8-146 PMC4015352

[12] Nascimento RM , Otto PA , de Brouwer AP , Vianna‐Morgante AM . UBE2A, which encodes a ubiquitin‐conjugating enzyme, is mutated in a novel X‐linked mental retardation syndrome. Am J Hum Genet. 2006;79(3):549‐555. 16909393 10.1086/507047 PMC1559544

[13] Ma D , Tan J , Zhou J , et al. A novel splice site mutation in the UBE2A gene leads to aberrant mRNA splicing in a Chinese patient with X‐linked intellectual disability type Nascimento. Mol Genet Genomic Med. 2019;7(11):e976. 31566921 10.1002/mgg3.976 PMC6825863

[14] Budny B , Badura‐Stronka M , Materna‐Kiryluk A , et al. Novel missense mutations in the ubiquitination‐related gene UBE2A cause a recognizable X‐linked mental retardation syndrome. Clin Genet. 2010;77(6):541‐551. 20412111 10.1111/j.1399-0004.2010.01429.x

[15] Giugliano T , Santoro C , Torella A , et al. UBE2A deficiency in two siblings: a novel splicing variant inherited from a maternal germline mosaicism. Am J Med Genet A. 2018;176(3):722‐726. 29283210 10.1002/ajmg.a.38589

[16] Haddad DM , Vilain S , Vos M , et al. Mutations in the intellectual disability gene Ube2a cause neuronal dysfunction and impair parkin‐dependent mitophagy. Mol Cell. 2013;50(6):831‐843. 23685073 10.1016/j.molcel.2013.04.012

[17] Honda S , Orii KO , Kobayashi J , et al. Novel deletion at Xq24 including the UBE2A gene in a patient with X‐linked mental retardation. J Hum Genet. 2010;55(4):244‐247. 20339384 10.1038/jhg.2010.14

[18] Tolmacheva EN , Kashevarova AA , Nazarenko LP , et al. Delineation of clinical manifestations of the inherited Xq24 microdeletion segregating with sXCI in mothers: two novel cases with distinct phenotypes ranging from UBE2A deficiency syndrome to recurrent pregnancy loss. Cytogenet Genome Res. 2020;160(5):245‐254. 32485717 10.1159/000508050

[19] Tsurusaki Y , Ohashi I , Enomoto Y , et al. A novel UBE2A mutation causes X‐linked intellectual disability type Nascimento. Hum Genome Var. 2017;4:17019. 28611923 10.1038/hgv.2017.19 PMC5462939

[20] Wolanska E , Pollak A , Rydzanicz M , et al. The role of the reanalysis of genetic test results in the diagnosis of dysmorphic syndrome caused by inherited xq24 deletion including the UBE2A and CXorf56 genes. Genes (Basel). 2021;12(3):350. 33673493 10.3390/genes12030350 PMC7997426

[21] Arslan Satilmis SB , Kurt EE , Akcay EP , Sazci A , Ceylan AC . A novel missense mutation in the UBE2A gene causes intellectual disability in the large X‐linked family. J Gene Med. 2021;23(2):e3307. 33368912 10.1002/jgm.3307

[22] Hu H , Haas SA , Chelly J , et al. X‐exome sequencing of 405 unresolved families identifies seven novel intellectual disability genes. Mol Psychiatry. 2016;21(1):133‐148. 25644381 10.1038/mp.2014.193 PMC5414091

[23] Tucker T , Zahir FR , Griffith M , et al. Single exon‐resolution targeted chromosomal microarray analysis of known and candidate intellectual disability genes. Eur J Hum Genet. 2014;22(6):792‐800. 24253858 10.1038/ejhg.2013.248 PMC4023222

